# Virtual colonoscopy in stenosing colorectal cancer

**DOI:** 10.1186/1750-1164-3-11

**Published:** 2009-11-09

**Authors:** Marco Coccetta, Carla Migliaccio, Francesco La Mura, Eriberto Farinella, Ioanna Galanou, Pamela Delmonaco, Alessandro Spizzirri, Vincenzo Napolitano, Lorenzo Cattorini, Diego Milani, Roberto Cirocchi, Francesco Sciannameo

**Affiliations:** 1 Department of General Surgical, St Maria Hospital, Terni, University of Perugia, Perugia, Italy

## Abstract

**Background:**

Between 5 and 10% of the patients undergoing a colonoscopy cannot have a complete procedure mainly due to stenosing neoplastic lesion of rectum or distal colon. Nevertheless the elective surgical treatment concerning the stenosis is to be performed after the pre-operative assessment of the colonic segments upstream the cancer.

The aim of this study is to illustrate our experience with the Computed Tomographic Colonography (CTC) for the pre-operative assessment of the entire colon in the patients with stenosing colorectal cancers.

**Methods:**

From January 2005 till March 2009, we observed and treated surgically 43 patients with stenosing colorectal neoplastic lesions. All patients did not tolerate the pre-operative colonoscopy. For this reason they underwent a pre-operative CTC in order to have a complete assessment of the entire colon. All patients underwent a follow-up colonoscopy 3 months after the surgical treatment. The CTC results were compared with both macroscopic examination of the specimen and the follow-up coloscopy.

**Results:**

The pre-operative CTC showed four synchronous lesions in four patients (9.3% of the cases). The macroscopic examination of the specimen revealed three small sessile polyps (3 - 4 mm in diameter) missed in the pre-operative assessment near the stenosing colorectal cancer. The follow-up colonoscopy showed four additional sessile polyps with a diameter between 3 - 11 mm in three patients.

Our experience shows that CTC has a sensitivity of 83,7%.

**Conclusion:**

In patients with stenosing colonic lesions, CTC allows to assess the entire colon pre-operatively avoiding the need of an intraoperative colonoscopy. More synchronous lesions are detected and treated at the time of the elective surgery for the stenosing cancer avoiding further surgery later on.

## Background

The patients suffering from stenosing colorectal cancer are frequently treated for occlusion (20%) and stenosis (16%) [1]. The elective surgical treatment concerning the stenosis is to be performed after the preoperative assessment of the colonic segments upstream the cancer.

If the endoscopic instrument can pass over the lesion, the gold standard is the colonoscopy. On the other hand, when the instrument cannot surmount the stenosis, the proximal segment of colon is to be evaluated through a double contrast barium enema. This approach shows low sensitivity and specificity particularly in the right colon [2].

Threfore when barium enema does not lead to an accurate evaluation, the patient needs an intra-operatory colonoscopy [3]. This investigation implies a few difficulties as the patient needs to be positioned on the operating table. It also requires additional time and can make the abdominal closure more difficult, because of the bowel distension caused by the insufflated air [4,5].

When the endoscopic procedure is incomplete because of the presence of a severe stenosing lesion, a further colonoscopy is required at 3-month follow up [6,7], in order to rule out a missed synchronous lesions.

The recent introduction of CTC allows a more adequate preoperative assessment of colon showing higher sensitivity and specificity [8]. The aim of this study is to illustrate our experience with the CTC for the pre-operative assessment of the entire colon in the patients with stenosing colorectal cancer.

## Methods

From January 2005 till March 2009, we observed and treated 43 patients with stenosing colorectal neoplastic lesions presenting with changes in the bowel habit, constipation, abdominal pain and lower gastrointestinal bleeding. We observed 28 males and 15 females aged between 47 and 73 years (average age 65 years). All patients did not tolerate the endoscopic procedure. For this reason they underwent a CTC in order to have a complete pre-operative assessment of the entire colon. All 43 patients underwent a pre-operative CTC in order to have a complete assessment of the entire colon. All 43 patients underwent surgical resections and specimen was carefully evaluated to search for any synchronous lesions. Furthermore all patients underwent a follow-up colonoscopy at 3 months from surgery.

The results from CTC were compared with both macroscopic examination of specimen and the follow-up colonscopy.

## Results

The CTC showed synchronous lesions in 4 patients (9.3% of the cases) (Table 1):

**Table 1:** Stenosing colorectal cancer and synchronous lesions showed in the pre-operative CTC

**Site of stenosing colorectal cancer**	**Synchronous lesions Site**	**Synchronous lesion Type**	**Diameter of synchronous lesions**	**Surgical Treatment**
Sigmoid colon	Descending colon	Pedunculated polyp	8 mm	Left laparoscopic hemicolectomy
Rectum	Ascending colon	Pedunculated polyp	12 mm	Laparoscopic anterior resection of rectum
Sigmoid colon	Ascending colon	Sessile polyp	18 mm	Left laparotomic hemicolectomy and excision of polyp through enterotomy
Descending colon	Ascending colon	Voluminous vegetating cancer	36 mm	Subtotal laparotomic colectomy

• a pedunculated polyp in two cases (8 and 12 mm of diameter) both in the left colon;

• a sessile polyp (18 mm of diameter) in ascending colon;

• a voluminous vegetating lesion (36 mm of diameter) in the ascending colon.

The patients with synchronous colonic lesions are usually treated through a laparotomic access, as identifying laparoscopically synchronous lesions without a tattoo marked preoperatively is difficult.

Furthermore, often it is not possible to identify synchronous lesions by intra-operative palpation even if the diameter is larger than one centimetre [9,10]. This supports the need of good preoperative assessment.

In our series the patients with synchronous neoplastic lesions underwent the following surgical treatments (Table 1):

• Left laparoscopic hemicolectomy (stenosing sigmoid cancer with adjacent peduncalated polyp of descending colon)

• Laparoscopic anterior resection of rectum (stenosing rectal cancer with adjacent peduncalated polyp of sigmoid colon).

• Left laparotomic hemicolectomy associated with excision of polyp through enterotomy in the ascending colon (stenosing cancer of sigmoid colon associated a sessile polyp of ascending colon).

• Subtotal laparotomic colectomy for two synchronous neoplastic lesions located in distant colic segments (descending colon stenosing cancer associated with vegetant neoplastic lesion of ascending colon).

### Table 1

The macroscopic examination of the specimen revealed three patients with small sessile polyps (3 - 4 mm in diameter) missed in the pre-operative CTC near the stenosing colorectal cancer (Table 2).

**Table 2:** Stenosing colorectal cancers and synchronous lesionsidentified specimen and missed in the pre-operative CTC.

**Site of stenosing colorectal cancer**	**Site of synchronous lesion**	**Type of synchronous lesions**	**Diameter of synchronous lesions**
Left colon	Left colon	Sessile polyp	3 mm
Left colon	Left colon	Sessile polyp	4 mm
Left colon	Left colon	Sessile polyp	3 mm

### Table 2

The follow-up colonscopy showed four additional endoluminal lesions in three patients: four sessile polyps with diameter of 3, 4, 8 and 11 mm (Table 3)

**Table 3:** Stenosing colorectal cancers and synchronous lesions identified in the follow-up colonoscopy at 3 months after the surgical treatment and missed in the pre-operative CTC

**Site of stenosing colorectal cancer**	**Site of synchronous lesion**	**Type of synchronous lesions**	**Diameter of synchronous lesions**
Left colon	Ascending colon	Sessile polyp	3 mm
Left colon	Transverse colon	Sessile polyp	4 mm
Left colon	Transverse colon	Sessile polyp	8 mm
Rectum	Left colon	Sessile polyp	11 mm

### Table 3

Our experience shows that virtual colonoscopy has a sensitivity of 83,7%.

## Discussion

Computed Tomographic Colonography (CTC) is also known as "virtual colonoscopy" and it was introduced in 1994. It is considered a noninvasive method of imaging and it allows to explore the colon by employing a helical CT [11].

Data from literature show that CTC is safer than colonoscopy. Indeed a colonic perforation occurs in 1:1000 patients undergoing conventional colonoscopy and mortality associated with this procedure is 1:5000 [12-17], while the morbidity and mortality associated with CTC are similar to those of the air-contrast barium enema (perforation rate of 1:10000 and mortality rate of 1:50000) [18-20].

There is only one study in literature showing a perforation rate, which is very low, up to 0.04% (3/7180) [21] and no study showing a case of death after performing a CTC.

The CTC has recently been proposed as the standard investigation for the pre-operative assessment of the colonic segments proximal to the obstructive cancer, being well tolerated, less invasive and showing good results [4,22-25].

Usually the colonoscopy is not fully carried out up to the ileocecal valve in 5-10% of the patients; this is mainly due to [26]:

• presence of stenosing neoplastic lesion of rectum or distal colon (58,3%):

• presence of dolicocolon (33%);

• presence of spastic colon (11,7%).

• presence of extrinsic compressions (1,6%);

• presence of intestinal malrotation (1,6%);

• presence of visceral adhesions.

In the past in all these cases the alternative investigation was an opaque enema. Nowadays, CTC shows a higher sensitivity making it the choice investigation when the traditional endoscopy cannot be carried out satisfactorily [26].

All CTCs performed in the above mentioned cases were successful (91,7-100%) (table 4) [4,22,23,27].

**Table 4:** Virtual colonoscopy in stenosing colorectal cancers: sensitivity for synchronous lesions after colonoscopy follow-up at 3 months

		**Preoperative evaluation of the colon proximal to the obstructive colorectal cancer**	**Pts. with colorectal cancers and obstructing colorectal lesions**	**Lesions missing at preoperative virtual colonoscopy**	**Sensitivity**
Fenlon	1999	CTC	12 pts.	2	83.4%
Galia	2001	CTC	19 pts.	3	83.4%
Morrin	2005	CTC	17 pts	1	93%
Nagata	2009	PET/CT colonography	13 pts.	0	100%

### Table 4

The cause of failure is frequently based either on neoplastic obstruction or on an inadequate air distension of proximal colon (8,3%) [26].

Data from literature show that CTC allows the diagnosis of synchronous lesions in 27,2%-89,6% of cases [28]. CTC substantially owns the same sensitivity as an optical colonoscopy for polyps > or = 7 mm in diameter [29]. Most of the polyps were observed in ascending colon (31,25%), transverse colon (18,75%), descending colon (18,75%) and sigma (6,25%) [26]. The diameter of the polyps varies between 5 mm (25%), 6-9 mm (62,5%) and 10 mm (12,5%) [26]. Between 6.25% and 8.3% of the observed synchronous lesions resulted to be cancers [22,26].

In those patients with synchronous cancers the most appropriate approach is to remove both lesions at the same time. Fenlon et al. described a subtotal colectomy for sigmoid and trasverse colon cancers and a left hemicolectomy for spleen flexure and sigma lesion [22].

Mingyue et al stated in their study that although the synchronous lesion is a polyp, it should be removed during surgery, otherwise, in the remaining cases, polyps will be removed during the endoscopic follow-up [26].

In the past, a considerable percentage of CTC presented a high incidence of false positives (between 54.6% and 64% for polyps within 6-10 mm of diameter, and 41-51% for polyps larger than 10 mm in diameter) [10,30,31].

Nowadays more modern softwares and new methods for colon cleansing preparation allow a significant reduction of false positives [32], lowering the percentage till 2.1% for polyps and 0.7% for cancers [33] despite the first results appeared in literature [34-36].

Moreover the possibility to perform the CTC even with a mild bowel preparation allows to extend the indication of the cancer screening to those patients considered not enough fit to tolerate a complete bowel preparation [37].

**Figure 1:**
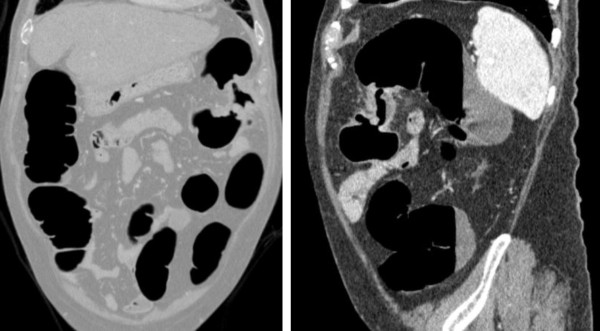
**Cancer of splenic flexure unsurpassed by colonoscopy**.

**Figure 2:**
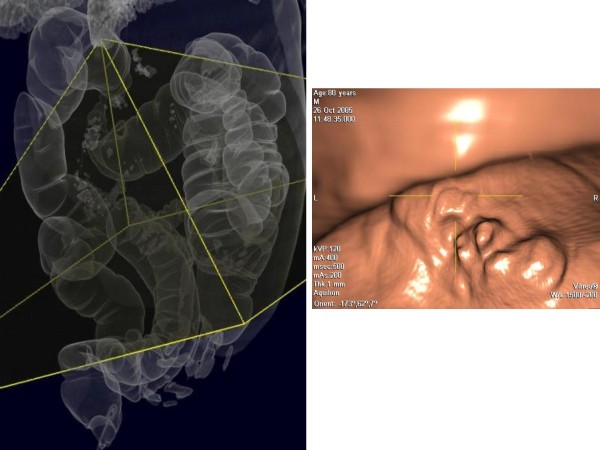
**3D Reconstruction with iconographic effect similar to double contrast barium enema of splenic flexure's cancer**.

**Figure 3:**
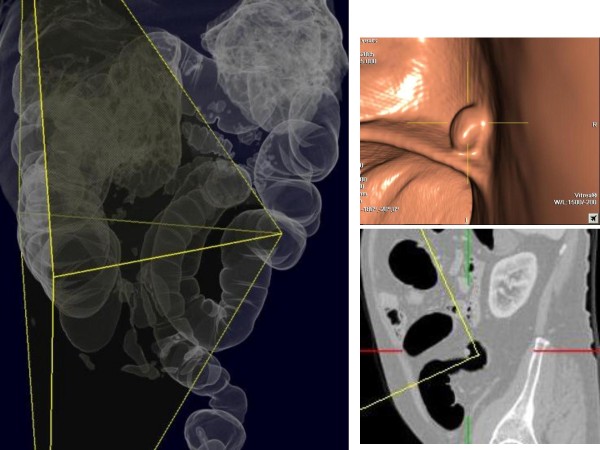
**Downstream polyp of splenic flexure cancer's**.

**Figure 4:**
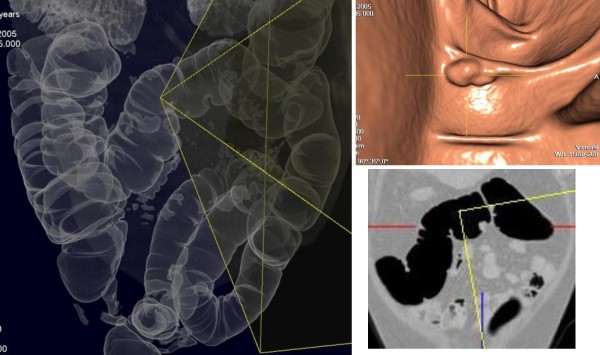
**Upstream polyp of splenic flexure cancer's**.

An accurate evaluation of the colonic segments upstream the neoplastic obstruction is extremely important as the patients with colorectal cancer are often affected by synchronous neoplastic lesions (villous adenomas in 33-55% of the cases and other types of cancers in 3% of the cases) [36,38,39] (figures 1,2,3,4). A surgical re-intervention is required when synchronous neoplastic lesions are detected and this increases morbidity and higher complication rates, especially because of peritoneal adhesions.

In case of synchronous lesions the prognosis becomes worse than a single cancer (55% survival at 5 years) [40], especially when diagnosis and treatment are not carried out on time.

Currently CTC is very helpful in colorectal surgery and it is particularly useful during the preoperative study in laparoscopic colorectal surgery; an accurate localization of the lesions not only allows an appropriate trocar insertion, but it also reduces the error due to a lack of tactile sensation [41].

It can be difficult to find out whether synchronous colon cancers are present in an intestinal segment proximal to the obstructive cancer. These patients usually need a further sequent surgical treatment if synchronous colon cancers are detected later [42]. An accurate diagnosis including the evaluation of the colonic segments proximal to the obstruction can avoid additional surgical approaches providing a better quality of life, as well as being cost-effective [9,43-45]. Therefore, one of the important roles of preoperative diagnosis in patients with obstructive cancer is a precise evaluation of the entire colon [4].

## Conclusion

This study shows CTC as a valid pre-operative investigation (sensitivity 83.7%) in order to detect synchronous neoplastic lesions in the patients who do not tolerate the conventional colonoscopy because of stenosing colorectal cancer. The main benefits from from its employment are that the intraoperative colonoscopy is not needed and more synchronous lesions are detected and treated at the time of the elective surgery for the stenosing cancer avoiding further surgery later on.

## Competing interests

The Authors state that none of the authors involved in the manuscript preparation has any conflicts of interest towards the manuscript itself, neither financial nor moral conflicts. Besides none of the authors received support in the form of grants, equipment, and/or pharmaceutical items.

## Authors' contributions

All authors contributed equally to this work.

## Consent

Written informed consent was obtained from the patient for publication of this case report and accompanying images. A copy of the written consent is available for review by the Editor-in-Chief of this journal.
